# Weight self-stigma, weight bias internalization, and eating attitudes in relation to body appreciation

**DOI:** 10.3389/fpsyg.2026.1772302

**Published:** 2026-02-25

**Authors:** Tevfik Koçak, Emine Kocyigit, Yagmur Demirel Ozbek, İsa Celik

**Affiliations:** 1 Department of Nutrition and Dietetics, Faculty of Health Sciences, Gümüşhane University, Gümüşhane, Türkiye; 2 Department of Nutrition and Dietetics, Faculty of Health Sciences, Ordu University, Ordu, Türkiye; 3 Department of Nutrition and Dietetics, Faculty of Health Sciences, Recep Tayyip Erdoğan University, Rize, Türkiye; 4 Department of Nursing, Faculty of Health Sciences, Recep Tayyip Erdoğan University, Rize, Türkiye

**Keywords:** eating attitudes, weight bias internalization, body appreciation, weight self-stigma, young adults

## Abstract

**Objective:**

This study aimed to investigate the relationships between weight self-stigma, weight bias internalization, eating attitudes, and body appreciation in young adults, and to ascertain the distinct impact of these variables on body appreciation.

**Methods:**

A cross-sectional study was carried out including young adults aged 19 to 35 years, comprising 69.6% females and 30.4% males. Participants completed a questionnaire evaluating weight self-stigma, internalization of weight bias, eating attitudes, and body appreciation. Hierarchical multiple regression analyses were used to assess the additional impacts of weight-related stigma and eating attitudes on body appreciation, while adjusting for demographic and anthropometric factors.

**Results:**

Weight self-stigma, particularly the internalization of weight bias, showed a significant negative association with body appreciation. Weight self-stigma and its subdimensions (self-devaluation and fear of enacted stigma) had substantial negative correlations with body appreciation. These correlations encompassed the subdimensions of body appreciation, specifically general body appreciation and body image investment. While eating attitudes did not show a significant correlation with body appreciation at the bivariate level, they became a significant predictor in the hierarchical regression model, suggesting a conditional (suppression) effect after accounting for weight-related stigma and BMI.

**Conclusion:**

The findings emphasize the important function of weight-related stigma in diminishing body appreciation among young adults and stress the necessity of examining eating attitudes in conjunction with stigma-related factors. Interventions designed to enhance positive body image should focus on both internalized weight stigma and maladaptive eating-related attitudes.

## 1 Introduction

Body image is a multifaceted and variable construct encompassing a person’s thoughts, feelings, and behaviors related to their body (Tylka and Wood-Barcalow, 2015). This construct encompasses not only evaluations related to physical appearance, but also the value a person places on their body, their attitude toward their body, and the emotional bond they have established with their body (Torres et al., 2024). Previous research on body image has mostly focused on risk-oriented issues such as body dissatisfaction, body shame, and negative body image (Lacroix et al., 2023; Merino et al., 2024). Consequently, the literature has primarily focused on pathological and deficit-oriented outcomes, with insufficient emphasis on protective and resilience-focused aspects of body image and their potential contribution to enhancing psychological wellbeing. However, in recent years, approaches based on positive and protective factors related to body image have gained more importance to address this gap (Önal, 2025; Tort-Nasarre et al., 2023). The concept of positive body image developed in this context involves individuals not evaluating their bodies solely based on aesthetic criteria, appreciating their bodies’ functionality, and taking a critical stance against unrealistic body ideals imposed by society (Cerea et al., 2025; Tylka and Wood-Barcalow, 2015; Wood-Barcalow et al., 2024).

Body appreciation, one of the important components of positive body image, is defined as the individual’s acceptance of their body as it is, embracing their physical differences without judgment, and developing an attitude of respect and compassion toward their body (Deveci et al., 2025; Matera et al., 2024). There are many factors that can influence body appreciation. These factors include the individual’s social environment and various psychosocial stressors they are exposed to (Hanson et al., 2024). A systematic review and meta-analysis showed that body appreciation was positively related to psychological wellbeing, self-esteem, and self-compassion; it is negatively related to negative psychological outcomes such as depressive symptoms, body shame, and body dissatisfaction (Linardon et al., 2022). Prior studies indicated that body image-related factors, such as body appreciation, weight stigma, and eating attitudes, exhibited gender-specific differences (He et al., 2020; Saunders et al., 2022). Women generally displayed diminished body appreciation, heightened body dissatisfaction, and more pronounced correlations among stigma, shame, and disordered eating, especially when associated with an elevated body mass index (BMI) (Argyrides et al., 2025; Aruna et al., 2024). This highlights the necessity for preventive and intervention strategies that are attuned to gender and weight-related disparities. This research collectively indicates that body appreciation is integrated within wider social and psychosocial frameworks, rather than existing as an isolated individual construct.

Current evidence shows that body appreciation is not solely an individual phenomenon but is influenced by wider cultural and psychosocial variables, with weight-based stigma being a notably significant element (Hanson et al., 2024; Linardon et al., 2022). Furthermore, a major social factor that harms body image in modern societies is weight-based stigma (Clark et al., 2021). Weight stigma refers to people being subjected to negative stereotypes, facing discrimination, and being socially ostracized due to their body weight or shape (Fruh et al., 2021). The effects of these adverse experiences on individuals extend beyond external exposure, intensifying and becoming more enduring as individuals internalize the negative societal messages directed at them (Nutter et al., 2024). In this context, internalized weight bias denotes the phenomenon in which an individual internalizes negative stereotypes related to weight and assesses themselves according to these biases. Internalized weight bias is a primary psychological process that adversely affects an individual’s self-perception and body image, with its associated with reported to be independent of BMI (Pearl and Puhl, 2018). Weight self-stigma, closely associated with internalized weight bias, involves the emergence of adverse emotions, including shame, guilt, and self-deprecation, stemming from an individual’s body weight (Nutter et al., 2024). Reports indicate that the subdimensions of weight self-stigma, notably “self-devaluation” and “fear of stigmatization,” significantly affect an individual’s body image and psychological health (Romano et al., 2022). The existing literature demonstrates that weight self-stigma and internalized weight bias correlate with adverse mental health outcomes, including depression, anxiety, and diminished self-esteem (Emmer et al., 2020; Fields et al., 2021). Moreover, it is underscored that these two concepts are interconnected psychological processes that ought to be assessed concurrently (Pearl and Puhl, 2018; Romano et al., 2022). While weight self-stigma and weight bias internalization are conceptually related, they represent partially separate psychological mechanisms. Weight self-stigma specifically highlights self-imposed devaluation and anticipated stigma, while weight bias internalization encompasses the wider internal acceptance of society weight-related stereotypes. Simultaneously analyzing both constructs could provide a more thorough comprehension of weight-related stigma mechanisms. Current findings indicated that females exhibited a higher tendency to internalize weight-related stereotypes, and this internalization serves as a more significant predictor of body-focused shame, psychological distress, and disordered eating behaviors (Lin et al., 2023; Yangyuen et al., 2025). In contrast, males mainly experienced weight-related stigma linked to ideals of body size, strength, or muscularity, marked by quite distinct emotional and behavioral patterns (Just-Nørregaard et al., 2025; Lin et al., 2023). These findings showed that gender significantly influenced the pathways through which internalized weight stigma impacted body image and broader psychological impacts. Furthermore, internalized weight-related stigma was found to affect eating attitudes, as people who internalized such stigma demonstrated increased levels of restrictive eating, emotional eating, and loss-of-control eating behaviors (Calugi et al., 2023; Levinson et al., 2024; Nutter et al., 2024; Saunders et al., 2022). In this context, eating attitudes are conceptualized as behavioral expressions of internalized weight-based stigma, reflecting how individuals’ experiences of stigma are expressed in eating-related thoughts and behaviors in daily life (Durso and Latner, 2008). From a theoretical standpoint, weight stigma, internalized weight bias, eating attitudes, and body appreciation can be positioned within a shared psychosocial framework in which external weight-based stigma is internalized and subsequently reflected in eating-related behaviors and body image outcomes.

While body image is often conceptualized as a protective factor against maladaptive eating behaviors, emerging theories suggest that eating attitudes may also reflect behavioral changes stemming from internalized weight-related stigma. From this perspective, cognitions related to eating disorders can emerge alongside a positive body image, potentially leading to weight loss, by reinforcing self-evaluation processes shaped by stigma and body-related self-judgment. Accordingly, this study examines eating attitudes as a simultaneous psychosocial correlation of body image within a stigma-based framework, rather than establishing a one-way or causal relationship.

A review of the existing literature indicates a lack of studies that have concurrently evaluated weight self-stigmatization, weight bias internalization, eating attitudes, and body appreciation. Numerous studies have investigated the relationships among these variables at a bivariate level. The present study aimed to examine the hypothesized links among weight self-stigmatization, weight bias internalization, eating attitudes, and body appreciation in young adult males and females, based on prior empirical research. It was hypothesized that increased weight self-stigma and internalized weight bias would be associated with lower body appreciation, whereas eating attitudes would demonstrate a weaker association with body appreciation. This study employed a cross-sectional, correlational design to identify statistical relationships rather than establish causality. This approach may elucidate the relationship between these characteristics and body appreciation, fostering a more holistic understanding of body image and eating-related processes by examining them concurrently. By evaluating these constructs together, the present study addresses this gap and contributes to the literature by framing them within a single, comprehensive theoretical framework.

## 2 Materials and methods

### 2.1 Study design and participants

This cross-sectional and descriptive study was completed between April 15, 2025, and September 15, 2025. Each participant was informed of the inclusion and exclusion criteria for study participation (inclusion: ages 19–35; exclusion: mental disorders, long-term diseases, psychological disorders, eating disorder diagnoses, pregnancy, breastfeeding, and receiving of nutrition education), the study’s objectives, the time commitment required, and the procedures to be adhered to. The study was conducted in accordance with the Declaration of Helsinki. Participants provided informed consent.

The study included 878 people aged 19 to 35, comprising 611 females (69.6%) and 267 males (30.4%). Participants were willingly recruited using a convenience sampling method. Participants were recruited through announcements in university and community settings, and all interested individuals were briefed on the study’s objectives and methods before participating. The study was promoted as an investigation into body image and eating-related attitudes among young individuals. The majority of invited persons consented to participate; however, specific data on refusal rates were not consistently documented. Data were collected via self-report questionnaires, and in-person interviews were conducted to assist participants throughout the study. The average time taken for completing the questionnaires was about 15 min. *A priori* power analysis was performed utilizing GPower (version 3.1; F tests, linear multiple regression: fixed model, *R*^2^ increase), with *α* established at 0.05 and statistical power at 1 − *β* = 0.95. Assuming a small incremental effect size (*f*^2^ = 0.021) for the tested block (*u* = 2) in a model comprising six variables, the necessary sample size was approximately *N* = 750, aligning with previous extensive research on weight-related stigma and body image (Styk et al., 2024). However, more participants were incorporated into the study due to the potential for withdrawal. The research was conducted with 878 young adults.

### 2.2 Measures

#### 2.2.1 Sociodemographic and anthropometric characteristics

The researchers used questionnaires and in-person interviews to support participants in the study. The fundamental data form asked questions about having chronic diseases, current smoking status, consumption of alcohol, number of main meals and snacks, and sociodemographic factors (gender, age [year], grade of education, and monthly income). Individuals’ body weight and height were recorded based on self-reported data. The formula for calculating the BMI was BMI (kg/m^2^) = weight in kilograms/height in meters. The BMI values were classified according to the criteria established by the World Health Organization (Khanna et al., 2022).

#### 2.2.2 Weight self-stigma questionnaire (WSSQ)

The Weight Self-Stigma Questionnaire (WSSQ) requests participants to evaluate their perception of weight-related self-stigma (Lillis et al., 2010). The Turkish validity and reliability of the scale were conducted by Sevincer et al. (2017). The WSSQ is a 12-item instrument evaluating weight-related self-stigma, consisting of two 6-item domains: self-devaluation and fear of enacted stigma. Items 1 to 6 form the self-devaluation subscale, whereas items 7 through 12 comprise the enacted labeling fear subscale. One example from the WSSQ is: “I feel guilty because of my weight problems.” A five-point Likert scale, ranging from 1 (strongly disagree) to 5 (strongly agree), is used to score each of the 12 items. The overall WSSQ score is derived by summing all questions, where elevated scores signify increased weight-related self-stigma. The reliability analysis for the scale reveals a Cronbach’s Alpha coefficient of 0.74 for self-devaluation, 0.81 for the fear of labeling subscale, and 0.83 for the overall scale (Sevincer et al., 2017).

#### 2.2.3 Modified weight bias internalization scale (M-WBIS)

The modified weight bias internalization scale (M-WBIS), developed by Pearl and Puhl (2014), assesses internalized stigma, individuals’ perceptions of weight-related stigma across body weight classifications (Pearl and Puhl, 2014). The scale was translated into Turkish by Apay et al. (2017). The scale comprises 11 items and utilizes a 7-point Likert scale. An example item from the scale is: “I feel anxious about my weight because of what people might think of me.” Responses are recorded using a 7-point Likert scale, with options ranging from 1 (strongly disagree) to 7 (strongly agree). Elevated scores on the M-WBIS signify that an individual’s internalized weight biases are more pronounced in a negative manner. The scale showed high internal consistency (Cronbach’s alpha = 0.92) (Apay et al., 2017).

#### 2.2.4 Body appreciation scale (BAS)

The body appreciation scale (BAS) was created by Avalos et al. (2005) to assess an individual’s satisfaction with their body, acceptance of their body in its current state, and commitment to its care (Avalos et al., 2005). Scoring utilizes a 5-point Likert-type scale (1 = Never, 5 = Always), where higher total scores on this 13-item scale reflect increased body appreciation. One example item from the scale is: “I feel good about my body.” The examination on the validity and reliability of the scale in Turkish was conducted by Bakalım and Taşdelen-Karçkay (2016). There are two subscales and nine items in the Turkish version of the scale. Items 1, 2, 3, 4, 5, 8, and 9 indicate the first factor: General Body Appreciation, whereas items 6 and 7 indicate the second factor: Body Image Investment. There are no items with negative ratings; elevated scores indicate high body satisfaction. The Cronbach’s alpha coefficient for the scale was recorded as 0.94 in the Turkish version (Bakalım and Taşdelen-Karçkay, 2016).

#### 2.2.5 Eating attitude test (EAT-26)

The eating attitude test-26 (EAT-26), originally a 40-item scale developed in 1979, was revised and condensed to 26 items by Garner et al. (1982) to evaluate eating attitudes and behaviors (Garner et al., 1982). The scale’s validity and reliability were investigated in Türkiye by Ergüney-Okumuş and Sertel-Berk (2019). An example item from the scale is: “I avoid eating when I am hungry.” Items 1–25 are scored according to the standard EAT-26 scoring procedure, whereby responses of “Always,” “Usually,” and “Often” are assigned scores of 3, 2, and 1, respectively, while all other response options are scored as 0. Item 26 is reverse-scored, with “Never” assigned a score of 3 and decreasing values for more frequent responses. The total EAT-26 score is calculated by summing the item scores; higher scores indicate greater levels of disordered eating attitudes. The Turkish version of the scale exhibited a Cronbach Alpha internal consistency coefficient of 0.84 and a test–retest reliability coefficient of 0.78 (Ergüney-Okumuş and Sertel-Berk, 2019).

### 2.3 Data analysis

All analyses were conducted utilizing the Statistical Package for the Social Sciences (IBM SPSS 26). The statistical analyses included descriptive analyses of all fundamental variables, followed by correlation analyses and hierarchical regression analysis to evaluate the explained variation in body appreciation. The normality of the data distribution was verified by visual approaches (histograms and probability plots) and studies of skewness and kurtosis; however, due to the substantial sample size, distributional assumptions were predominantly assessed based on skewness and kurtosis values. The general statistics were presented as mean and standard deviation for numerical variables, along with frequency and percentage for categorical variables. The Pearson correlation coefficient was employed to demonstrate the relationships among numerical variables. Hierarchical multiple regression analyses were used to examine the relationships among weight self-stigma, weight bias internalization, eating attitudes, and body appreciation. Age, gender (0 = female, 1 = male), and BMI were incorporated as control variables in the initial block. In later models, WSSQ and M-WBIS were included separately, followed by their concurrent inclusion with EAT-26 in the final model. Including eating attitudes in the final regression model is theoretically justified because their predictive significance may become apparent only after controlling for shared variance with related psychological components. Hierarchical regression was used to assess the unique contribution of eating attitudes to body appreciation, independent of weight-related stigma and BMI. The changes in explained variance (Δ*R*^2^) were analyzed to determine the additional contribution of each block. Statistical significance was established at *p* < 0.05.

## 3 Results

Table 1 shows the characteristics of the participants. The mean age of the participants was 24.34 years (SD = 4.08). Participants indicated an average educational attainment of 14.46 years (SD = 2.09). The mean BMI was 23.86 kg/m^2^ (SD = 3.87), with the majority of participants categorized as normal weight (61.9%), followed by overweight (26.9%), underweight (4.6%), and obese (6.6%). The majority of participants were single (79.5%) and unemployed (67.2%).

**Table 1:** Characteristics of the study sample (*n* = 878).

Characteristics	*n* (%)
Sex	
Female	611 (69.6)
Male	267 (30.4)
Education level	
High school and below	320 (36.4)
Bachelor’s degree	537 (61.2)
Graduate degree (Master’s/PhD)	21 (2.4)
Employment status	
Employed	288 (32.8)
Unemployed	590 (67.2)
Marital status	
Single	698 (79.5)
Married	180 (20.5)
Monthly income	
Income < expenses	254 (28.9)
Income = expenses	463 (52.7)
Income > expenses	161 (18.3)
Having chronic disease	
Yes	62 (7.1)
No	816 (92.9)
Smoking status	
Yes	289 (32.9)
No	589 (67.1)
Alcohol consumption	
Yes	117 (13.3)
No	791 (86.6)
BMI classification	
Underweight	40 (4.6)
Normal	543 (61.9)
Overweight	236 (26.9)
Obese	58 (6.6)

	Mean ± SD
Age (years)	24.34 ± 4.08
Years of education	14.46 ± 2.09
Number of main meals	2.32 ± 0.52
Number of snacks	1.52 ± 0.84
BMI (kg/m^2^)	23.86 ± 3.87

BMI, body mass index.

Table 2 presents the descriptive statistics for all research variables. The mean scores revealed moderate levels of weight self-stigma and weight bias internalization, along with comparatively high levels of body appreciation. The skewness and kurtosis values for all variables were within acceptable limits, indicating no significant departures from normality and supporting the application of parametric analysis.

**Table 2:** Descriptive statistics of the study variables (*n* = 878).

Variable	Mean	SD	Min	Max	Kurtosis	Skewness
1. WSSQ	20.06	8.21	6.00	60.00	1.84	1.31
2. Self-devaluation	11.59	5.32	1.00	30.00	−0.02	0.84
3. Fear of enacted stigma	8.48	3.87	1.00	30.00	5.92	2.26
4. M-WBIS	23.77	14.30	10.00	77.00	1.55	1.39
5. BAS	34.97	8.00	9.00	45.00	0.17	−0.76
6. General body appreciation	27.51	6.20	7.00	35.00	0.34	−0.84
7. Body image investment	7.49	2.15	2.00	10.00	−0.39	−0.64
8. EAT-26	10.93	10.16	0.00	66.00	3.98	1.73

BAS, body appreciation scale; EAT-26, eating attitudes test-26; SD, standard deviation; M-WBIS, modified weight bias internalization scale; WSSQ, weight self-stigma questionnaire.

**Figure 1:**
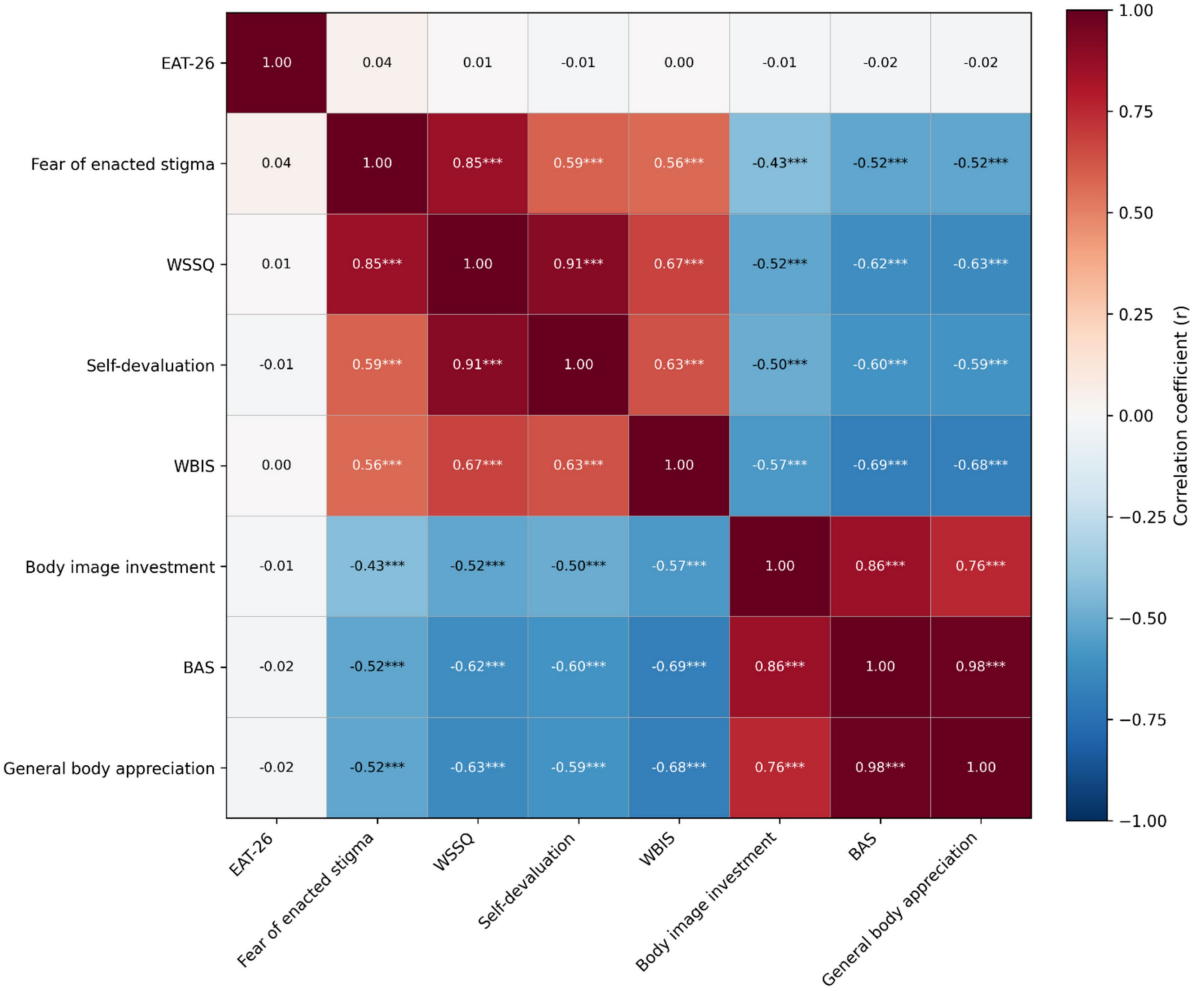
Pearson correlation matrix among study variables. The heatmap illustrates Pearson correlation coefficients among eating attitudes (EAT-26), weight-related stigma variables (Weight Self-Stigma Questionnaire [WSSQ], self-devaluation, fear of enacted stigma, and modified weight bias internalization scale [M-WBIS]), and body appreciation variables (Body Appreciation Scale [BAS], general body appreciation, and body image investment). Colors that are warm signify positive correlations, while cool colors denote negative correlations. Asterisks indicate statistically significant associations. BAS, body appreciation scale; EAT-26, eating attitudes test-26; M-WBIS, modified weight bias internalization scale; WSSQ, weight self-stigma questionnaire. ***p < 0.001.

As shown in Figure 1, Pearson correlation analyses indicated strong positive associations among weight-related stigma variables, including weight self-stigma, self-devaluation, fear of enacted stigma, and weight bias internalization. Weight-related stigma factors exhibited moderate to high negative correlations with body appreciation and its subdimensions, specifically general body appreciation and body image investment. Conversely, eating attitudes had no significant correlation with body appreciation characteristics at the bivariate level. Robust positive correlations were identified between total body appreciation and its subdimensions, affirming the internal consistency of the positive body image constructs.

Hierarchical regression analyses were performed to investigate the relationships among demographic characteristics, weight stigma, weight bias, eating attitudes, and body appreciation. Results are summarized in Table 3.

**Table 3:** Hierarchical regression models predicting body appreciation scale.

Variables	Model 1			Model 2			Model 3			Model 4		
	*B*	*SE*	*β*	*B*	*SE*	*β*	*B*	*SE*	*β*	*B*	*SE*	*β*
Age	0.08	0.05	0.05	0.09	0.08	0.05	0.09	0.08	0.05	0.09	0.08	0.05
Gender	0.14	0.42	0.01	0.05	0.14	0.01	0.04	0.14	0.01	0.04	0.14	0.01
BMI	−0.15	0.05	−0.15	−0.18	0.04	−0.17*	−0.16	0.04	−0.15*	−0.10	0.03	−0.09***
WSSQ				−0.34	0.03	−0.55***				−0.27	0.03	−0.33***
M-WBIS							−0.39	0.02	−0.63***	−0.27	0.02	−0.37***
EAT-26										−0.05	0.02	−0.05**
*R* ^2^	0.014			0.410			0.480			0.720		
Δ*R*^2^				0.396***			0.466***			0.240***		

Model 1 = Age, Gender, BMI. Model 2 = Model 1 + WSSQ. Model 3 = Model 1 + M-WBIS. Model 4 = Model 1 + WSSQ + M-WBIS + EAT-26. Dependent variable: BAS. Gender coded as 0 = female, 1 = male. BAS, body appreciation scale; BMI, body mass index; EAT-26, eating attitudes test-26; M-WBIS, modified weight bias internalization scale; WSSQ, weight self-stigma questionnaire. *p < 0.05, **p < 0.01, ***p < 0.001.

In Model 1, age, gender, and BMI were included as control variables. This model accounted for a minor percentage of the variance in body appreciation (*R*^2^ = 0.014). None of the predictors were statistically significant. The data indicated that, at the bivariate level, body appreciation did not substantially differ by gender.

In Model 2, WSSQ was incorporated into the model. The incorporation of WSSQ resulted in a significant enhancement in explained variance (Δ*R*^2^ = 0.396, *p* < 0.001). Increased levels of weight self-stigma were substantially correlated with diminished body appreciation, whereas BMI was also found to be a significant negative predictor. Gender remained to be a non-significant predictor following the incorporation of weight self-stigma.

In Model 3, M-WBIS was used in place of WSSQ. This model accounted for more of variance in body appreciation (*R*^2^ = 0.480), with M-WBIS exhibiting a significant negative correlation with BAS. BMI remained a significant predictor in this model. The effect of gender on body appreciation remained non-significant.

Finally, Model 4, including both weight stigma and weight bias with eating attitudes, explained a significant proportion of variance in body appreciation (*R*^2^ = 0.720). In this final model, both WSSQ and M-WBIS were substantial negative predictors of body appreciation, while eating attitudes exhibited a lower but statistically significant negative correlation. BMI remained inversely correlated with BAS, although age and gender were not significant predictors in any of the models. The results indicated that gender did not independently influence body appreciation when considering weight-related stigma and eating attitudes.

## 4 Discussion

Due to the cross-sectional and correlational nature of this study, all observed correlations must be considered as associative rather than causal. The findings demonstrate strong correlations among weight-related stigma, eating attitudes, and body appreciation; nevertheless, the directionality of these relationships remains indeterminate. In accordance with existing literature, these results are most accurately interpreted as indicative of concurrent psychological processes rather than direct consequences. This study examined the relationships among weight self-stigma, weight bias internalization, eating attitudes, and body appreciation in a large sample of young adults aged 19 to 35 years. The findings indicated that weight-related stigma—particularly weight bias internalization—was significantly associated with lower body appreciation after controlling for age, gender, and BMI. These results are consistent with previous research in young adult populations demonstrating strong links between weight-related stigma, internalized weight bias, and body image outcomes, such as body appreciation and psychological wellbeing (Alyami et al., 2025; Huang et al., 2024; Montague et al., 2025). Gender was a control variable in all analyses; however, it did not appear to be a significant predictor of body appreciation in any of the hierarchical regression models. This research indicates that the associated with weight-related stigma on body appreciation is significant regardless of gender. Despite gender not being a significant predictor of body appreciation in the current models, this result warrants careful interpretation. The lack of a primary effect of gender does not exclude the potential for gender to influence the strength of relationships among weight-related stigma, eating attitudes, and body appreciation. Previous research indicates that stigma-related mechanisms and body image experiences may function differently between genders, especially for societal expectations and weight-related standards. Future research utilizing interaction terms or moderation analyses is necessary to ascertain whether the strength of these connections varies between women and men.

Our study revealed a substantial negative association between weight-related self-stigma, including its subscales of self-devaluation and fear of enacted stigma, and body appreciation. Weight self-stigma impairs body appreciation by fostering internalized guilt and self-devaluation, which in turn diminishes accepting and respectful attitudes toward the body (Pearl et al., 2022; Zancu and Diaconu-Gherasim, 2024). The integration of negative weight-related beliefs into the self-concept related individuals to evaluate their bodies more critically and perceive them as flawed or undeserving of care (Davidsen et al., 2023; Romano et al., 2022). This process may undermine essential aspects of body appreciation, such as body acceptance and positive engagement with one’s physical self. The limited simultaneous application of the WSSQ and the BAS in published studies may reflect the ongoing conceptual divide between weight stigma research and the literature on positive body image. Studies on weight-related stigma have mainly focused on detrimental psychological consequences, including body dissatisfaction, shame, and disordered eating, highlighting risk and vulnerability mechanisms (Bidstrup et al., 2021; Cerolini et al., 2024). Recent studies demonstrated that weight-related stigma was strongly linked to adverse body-related outcomes, such as body dissatisfaction, shame, and disordered eating, highlighting its significance as a major psychosocial risk factor in adulthood (Puhl et al., 2021; Rubino et al., 2020). Conversely, positive body image constructs, such as body appreciation, have been framed within a strengths-based paradigm that highlights body acceptance, respect, and functionality, instead of just the absence of negative assessments relating to the body, such as body dissatisfaction and body shame (Toole et al., 2021).

Previous research demonstrated strong and comparable correlations among weight bias internalization, diminished positive body image, increased negative body image, and heightened self-devaluation (Austen et al., 2021; Meadows and Higgs, 2020; Pearl et al., 2021). In accordance with this literature, our findings demonstrated that elevated levels of weight bias internalization correlated with diminished body appreciation. Significantly, these adverse relationships extended beyond overall body appreciation to its subdimensions—general body appreciation and body image investment—indicating that weight-related stigma may compromise both general body acceptance and individuals’ involvement with their body image. The strong correlations observed between weight bias internalization and body appreciation raise important questions about the conceptual distinctiveness of weight bias internalization relative to related constructs, such as body image and self-assessment. These associations indicate that internalized weight prejudice may closely align with other negative body-related attitudes in both theoretical and practical contexts (Romano et al., 2022). Consistent with prior research, the substantial overlap among measures of weight bias internalization, body image, and self-devaluation highlights the risk of a jangle fallacy. This refers to mislabeling closely related or partially overlapping psychological processes as distinct constructs, related to the erroneous identification of similar phenomena as separate variables (Meadows and Higgs, 2020; Romano et al., 2022). Nonetheless, the extent of these relationships did not imply total redundancy, indicating that weight bias internalization possesses a certain level of construct-specific variance. Although weight bias internalization overlaps with general body-related self-evaluations, it captures weight-specific internalized stigma that is not fully accounted for by global body image or self-esteem. Therefore, future studies need to account for body image and self-esteem to elucidate the distinct associated with weight bias internalization beyond common self-evaluative mechanisms.

The negative associations identified between weight-related stigma variables and body appreciation are further supported by evidence indicating that internalized stigma processes adversely affect adaptive body-related self-evaluations. Previous studies indicated that internalized weight stigma correlates with reduced body acceptance and poorer body-related wellbeing, even when accounting for BMI (Abdoli et al., 2024; Lee et al., 2021; Pearl and Puhl, 2018). Furthermore, the internalization of weight bias has been associated with maladaptive self-focus and appearance-based self-worth, potentially elucidating its strong relationship with reduced body appreciation (Himmelstein et al., 2017). Body appreciation involves accepting and respecting one’s body, regardless of its size or shape (Aimé et al., 2020). Nonetheless, the present study revealed that BMI had a significant negative effect on body appreciation. This apparent disparity may reflect the influence of overarching sociocultural factors and stigma, where increased physical weight is frequently associated with negative social evaluation and internalized weight-related beliefs. Individuals with a higher BMI may struggle to accept their bodies in environments that prioritize thinness. Consequently, while body appreciation represents an ideal of size-independent acceptance, its expression may still be affected by individual experiences of weight-related stigma and sociocultural constraints about body weight.

Eating attitudes were identified as a small yet statistically significant predictor in the hierarchical multiple regression model, although they were not significantly associated with body appreciation at the bivariate level. The inclusion of these variables in the final regression model was theoretically justified and enabled the identification of a potential conditional or suppression effect. In this context, eating attitudes explained unique variance after accounting for shared variance with weight-related stigma variables and BMI. Recent applied psychology research utilizing hierarchical regression frameworks has increasingly recognized such effects, especially in the analysis of connected psychosocial factors (Chiou et al., 2023; Vogel et al., 2025). After controlling for weight-related stigma factors and BMI, the present study found only a modest, incremental statistical contribution of eating attitudes to body appreciation, rather than a primary or directional influence. The literature primarily conceptualizes body appreciation as a protective factor that is inversely associated with disordered eating tendencies. Consequently, these findings indicate a limited, context-dependent association rather than supporting the notion that eating attitudes are a principal determinant of body appreciation (Baceviciene and Jankauskiene, 2020; Mallaram et al., 2023). The lack of gender as a significant predictor may be attributed to the predominance of female participants in the sample or the more influential associated with variables such as weight-related stigma and eating attitudes on body satisfaction. This example indicates that, in specific contexts, psychosocial processes related to weight may take precedence over gender differences.

A major strength of this study is its substantial sample size, which afforded sufficient statistical power to investigate the distinct and collective effects of weight-related stigma and eating attitudes on body appreciation. Examining both general body appreciation and its subdimensions facilitated a more comprehensive comprehension of positive body image processes. The utilization of hierarchical multiple regression signifies a methodological advantage, as it facilitated the evaluation of the additional explanatory contribution of stigma- and eating-related variables beyond demographic and anthropometric components. This study has certain limitations. The cross-sectional methodology prevents causal inferences about the directionality of the relationships among weight-related stigma, eating attitudes, and body appreciation. Theoretical models indicate that weight stigma may detrimentally affect positive body image, however reciprocal links cannot be dismissed. Secondly, all variables were evaluated by self-report measures, which may be susceptible to response biases and shared method variance, notwithstanding the utilization of approved instruments. Thirdly limitation of the current study is the exclusion of sexual orientation as a variable of investigation. Evidence suggests that body appreciation, experiences of weight stigma, and attitudes toward eating differ between heterosexual and sexual minority communities, highlighting variations in societal norms, exposure to minority stress, and ideals connected to body image (Fowler et al., 2025; Santoniccolo et al., 2025). Previous studies indicate that sexual minority individuals may exhibit unique patterns of body image worries, internalized stigma, and eating-related thoughts in contrast to heterosexual individuals, highlighting sexual orientation as a significant psychosocial variable in body image research (Convertino et al., 2021; de Tena and Badenes-Ribera, 2025). Therefore, the current findings must be taken cautiously, as unmeasured variability associated with sexual orientation may have impacted the observed correlations. Future research should clearly evaluate sexual orientation to clarify the mechanisms of weight-related stigma and positive body image across various sexual identity groups. Finally, the identified conceptual overlap among weight self-stigma, weight bias internalization, and body image-related factors necessitates careful interpretation. While the hierarchical regression models demonstrated distinct contributions of these variables to body appreciation, future research employing longitudinal or latent-variable methodologies is necessary to better clarify shared and construct-specific variation. The predominance of female participants in the sample restricts the generalizability of the findings to male and other gender groups. Despite controlling for gender in the results, future research should investigate the associations between weight-related stigma, eating attitudes, and body image utilizing more balanced samples or gender-specific analyses.

## 5 Conclusion

This study emphasizes the significance of weight-related stigma in comprehending body appreciation in young adults. The results indicate a strong correlation between weight self-stigma and weight bias internalization with diminished body appreciation, even when controlling for demographic variables, body mass index, and eating behaviors. Furthermore, eating attitudes distinctly influenced body appreciation when analyzed through a hierarchical regression model, highlighting the complex nature of positive body image. Collectively, these findings underscore the necessity for interventions and preventive measures that tackle both weight-related stigma and maladaptive eating-related cognitions to foster healthier and more favorable body image outcomes. These findings indicate that interventions designed to improve body image among youth should extend beyond weight- or appearance-focused methods and directly address internalized weight stigma and self-critical attitudes. Mental health professionals and healthcare providers should enhance prevention and therapeutic programs by integrating stigma-reduction strategies and body-acceptance components, rather than relying exclusively on weight control or dietary restriction.

## Data Availability

The raw data supporting the conclusions of this article will be made available by the authors, without undue reservation.
